# Comparative outcomes of synthetic and biological mesh use in laparoscopic inguinal hernia repair: a systematic review and meta-analysis

**DOI:** 10.1186/s12893-025-03151-w

**Published:** 2025-10-08

**Authors:** Candela Romano, Hugo Silva, Laura A. Gray, Carla Ibarra, William Soto, Lorenzo G. Fernandez, Jorge Vazquez del Real, Rafael Pinto-Colmenarez, Victor Sebastian Arruarana, Daniela Fulginiti

**Affiliations:** 1 University of California, Irvine, 101 The City Drive South, ZC4482, Orange, CA 92868 USA; 2 Universidad Autónoma de Baja California, Baja California, México; 3 Universidad Autónoma de Guadalajara, Tabasco, México; 4 Pontificia Universidad Católica del Ecuador, Quito, Ecuador; 5 Instituto Tecnológico de Santo Domingo, Santo Domingo, Dominican Republic; 6 Universidad Nacional de Córdoba, Córdoba, Argentina; 7 Universidad de Guadalajara, Guadalajara, Jalisco México; 8 Department of Ophthalmology, University of North Carolina at Chapel Hill, Chapel Hill, USA; 9 Brookdale Hospital, New York, NY USA; 10 Universidad Católica de Argentina, Buenos Aires, Argentina

**Keywords:** Inguinal hernia, Laparoscopic, Laparoscopic incisional hernia repair, Surgical mesh, Biological mesh, Synthetic mesh

## Abstract

**Background:**

Inguinal hernias occur when abdominal contents protrude through the inguinal canal. Laparoscopic repair is often preferred due to reduced postoperative pain, quicker recovery, and better cosmetic results. While synthetic mesh remains the standard, concerns about complications have prompted interest in biological meshes, which may integrate better with tissue but are more expensive and lack long-term data.

**Methods:**

We conducted a meta-analysis of studies published between 2015 and 2025 that compared biological versus synthetic mesh in laparoscopic inguinal hernia repair among adults. Only randomized controlled trials and cohort studies published in English were included. The primary and secondary outcomes were hernia recurrence and postoperative complications.

**Results:**

Out of 6017 records, three studies involving 1372 participants met the inclusion criteria. All compared porcine small intestinal submucosa (SIS) biological mesh with synthetic mesh. SIS mesh was associated with longer operating times (SMD 0.45; 95% CI: 0.02–0.87; *p* = 0.039). However, there were not a statistically significant differences in recurrence (RR 12.73; *p* = 0.15), complications (RR 3.06; *p* = 0.55), or adverse events (RR 5.38; *p* = 0.21). Heterogeneity was high, and funnel plots suggested possible publication bias.

**Conclusion:**

Biological mesh did not show a clear benefit over synthetic mesh in laparoscopic inguinal hernia repair. While it may reduce chronic pain, it requires longer operative time and raises concerns about cost and long-term outcomes. Larger, high-quality studies are needed to clarify their role in clinical practice.

**Supplementary Information:**

The online version contains supplementary material available at 10.1186/s12893-025-03151-w.

## Introduction

Inguinal hernias occur when abdominal contents protrude through a weak spot in the inguinal canal. They may arise from congenital or acquired factors, such as a patent processus vaginalis, aging, or chronic mechanical strain. The lifetime risk of developing an inguinal hernia is approximately 27% for men and 3% for women ​ [1]​. Diagnosis is generally clinical, and management ranges from observation in asymptomatic patients to surgical repair, most commonly with mesh reinforcement. Surgical repair is the definitive treatment for inguinal hernias. It is one of the most frequently performed surgical procedures worldwide, with over 700,000 repairs conducted annually in the United States​ [2]​. Both open and laparoscopic techniques are employed to achieve defect closure and tension-free repair. Laparoscopic approaches have become increasingly favored by surgeons due to their association with reduced postoperative pain, improved cosmetic outcomes, and recovery times shortened by 30-40%, all while maintaining comparable recurrence rates ​ [3]​. Mesh-based repair (hernioplasty) is the current standard, as it significantly lowers recurrence rates from 6-7% with primary tension repairs to around 2-3% when mesh is used. Traditionally, synthetic meshes have been the gold standard treatment due to proven long-term durability and cost-effectiveness ​ [4]​. However, concerns over foreign body reactions, chronic postoperative pain, and mesh-related complications have driven interest in alternative materials, particularly biological meshes derived from human or animal tissues. They are designed to integrate with host tissue, reducing inflammatory responses, particularly in contaminated fields ​ [5]​. Despite these potential advantages, the use of biological meshes in laparoscopic inguinal hernia repair remains controversial, largely due to higher costs (often 7 to 10 times greater than synthetic mesh) and lack of comprehensive long-term outcomes ​ [6]​. 

Despite the increasing application of biological mesh in various types of hernia repairs, there remains a lack of robust evidence directly comparing its performance to synthetic mesh in the context of laparoscopic inguinal hernia repair in non-contaminated cases. Given the unique considerations of the laparoscopic approach, this systematic review aims to evaluate the outcomes associated with synthetic versus biological meshes specifically in this setting. By focusing on recurrence rates, postoperative complications, chronic pain, infection, and cost-effectiveness, this review seeks to clarify the relative benefits and limitations of each mesh type, ultimately supporting more informed clinical decision-making for laparoscopic inguinal hernia repair. 

## Methods

This systematic review followed the recommendations and criteria established by the Preferred Reporting Items for Systematic Reviews and Meta-Analyses (PRISMA) ​ [7]​ reporting guidelines. The protocol was preregistered at the International Prospective Register of Systematic Reviews (PROSPERO) with the identifier code CRD420251031768. No separate detailed protocol document was prepared beyond the registration.

### Types of participants

The participant selection for this study included adults aged 18 and older who underwent laparoscopic inguinal hernia repair. Studies involving patients under the age of 18 were excluded.

### Types of interventions

The intervention group comprised participants who received biological mesh during laparoscopic inguinal hernia repair. The studies evaluated various biological materials derived from animal tissues to assess their efficacy in terms of surgical outcomes, recurrence, and complications. The comparator group included patients who underwent laparoscopic inguinal hernia repair using synthetic mesh.

### Outcomes

Our primary outcome of interest was hernia recurrence rate. Our secondary outcome considered was any postoperative complications, including infection, seroma, hematoma, mesh migration, and chronic pain where reported.

### Types of studies

This meta-analysis included studies that met the inclusion criteria: RCTs and cohort studies, published between 2015 and 2025 in English. We excluded case-control studies, case series, cross-sectional studies, dissertations, book chapters, protocol articles, reviews, news articles, conference abstracts, letters to the editor, editorials, and comment publications, along with articles on the pediatric population.

### Search methods

On April 4th of 2025, a systematic search was conducted on PubMed MEDLINE, Web of Science, Scopus, Cumulative Index to Nursing and Allied Health Literature, Cochrane, EMBASE, and Google Scholar using the search terms“Inguinal hernia OR inguinal”, “laparoscopic surgery OR laparoscopic”,“surgical mesh”, “biological mesh”, “synthetic mesh”, and “polypropylene”.

### Selection of studies

**Fig. 1:**
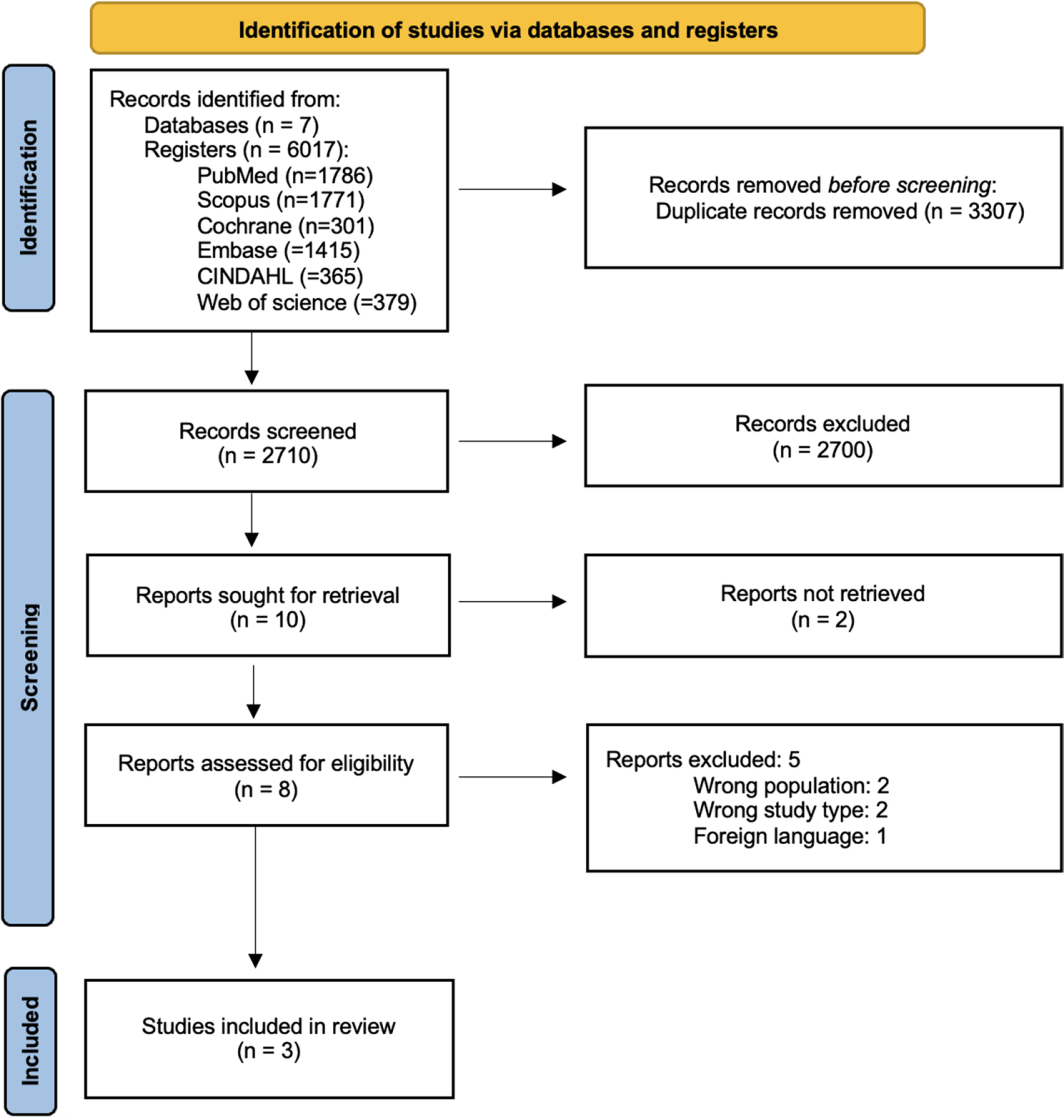
Prisma flow chart. This flow diagram outlines the systematic process of identifying, screening, and including studies in this meta-analysis

The study selection was achieved through Rayyan ​ [8]​ systematic review software. Duplicates were deleted, and then two reviewers screened the studies by title and abstract to exclude irrelevant studies. Subsequently, the studies were further analyzed by full-text assessment to ensure they met the established criteria and to discard any duplicates. The remaining articles were included for final analysis. In disagreements between reviewers, a third reviewer helped reach a consensus (Fig. 1).

### Assessment of risk of bias in included studies

To assess the quality of the studies included in the systematic review, we applied the Cochrane RoB 2.0 tool for RCTs and the Newcastle-Ottawa tool for cohort studies ​ [9, 10]​. Two independent reviewers evaluated the risk of bias in each study, and any discrepancies were resolved by discussion with a third, blinded reviewer.

### Data extraction

Two independent reviewers extracted the data, and disagreements were resolved by consensus. When multiple overlapping reports from the same study were identified, data from the report containing the most relevant information or from the first published report was included. Extracted data included sample sizes, intervention types, and measured outcomes. The outcomes comprised recurrence rates, complications, postoperative pain, and surgical site infections.

### Statistical analysis

A meta-analysis was performed using R version 3.4.3 (R Core Team)​ [11]​. The pooled effect of the outcomes was examined using a random-effects meta-analysis (DerSimonian-Laird approach) ​ [12]​. Whenever the number of studies reporting an outcome of interest was insufficient, only a qualitative analysis of the results was performed. Effect sizes were expressed as relative risk (RR) or standardized mean difference (SMD) and the 95% confidence interval. The I^2^ statistics assessed heterogeneity, and the following cut-off values were used for interpretation: <25, 25-50, and >50% were considered small, medium, and large heterogeneity, respectively. For all outcomes, sensitivity analyses using the leave-one-out method were performed to determine the influence of individual studies on the overall effect. Egger's regression test was used to examine publication bias when 10 or more reports with the same outcome were available. Whenever possible, subgroup analyses were performed for primary outcomes.

## Results

A total of 6,017 articles were identified through the initial search. After removing 3,307 duplicates, after screening 2,710 records, 2,700 were excluded based on title and abstract, leaving 10 studies for full-text assessment. After conducting a full-text assessment, we determined that two articles could not be retrieved ​ [13, 14]​, two did not cover the population of interest ​ [13, 15]​, two lacked the necessary data for inclusion ​ [16, 17]​, and one was published in a language other than English ​ [18]. ​In the end, three studies met the inclusion criteria and were included in the qualitative analysis ​ [19–21]​ (Fig. 1).

### Characteristics of included studies

Of the three studies included in the review, two were conducted in China​ [19, 20]​, and one in Taiwan ​ [21]​ Ho et al. (2015)​ [21]​ and Cuihong et al. (2025) ​ [20]​ were retrospective cohort studies, while Xue et al. (2024) ​ [19]​ was a RCT. All of them evaluated the use of porcine small intestinal submucosa (SIS) mesh compared to synthetic mesh in laparoscopic hernia repair. Patient populations varied, with mean ages ranging from 42.8 to 58.2 years, a balanced distribution of sex distribution across studies, and the follow-up period ranged from 18 to 84.2 months.

Operating time was consistently longer in the SIS mesh groups compared to controls in both retrospective cohorts, with modest differences of 78.9 vs 72.1 minutes ​ [21]​ and 63.2 vs 50.1 minutes ​ [20]​. Hospital stay was only reported by Ho et al. (2015) ​ [21]​, showing a slightly prolonged hospitalization for SIS cases (2.6 vs 1.7 days). Regarding recurrence, Ho et al. (2015) ​ [21]​ observed five cases in the SIS group, whereas the synthetic mesh control group had no such events. Consistent with these findings, Cuihong et al. (2025) ​ [20]​ also reported five recurrences exclusively in the SIS group, with none in the controls. Conversely, Xue et al. (2024) ​ [19]​ noted no recurrences among SIS patients, while two occurred in those who received synthetic mesh. In terms of complications, Ho et al. (2015) ​ [21]​ documented eight complications in the SIS group and eleven in the control group. Cuihong et al. (2025) ​ [21]​ reported five complications in the SIS group, with none in the control group. Xue et al. (2024) ​ [19] ​observed no complications in the SIS group and 6 in the control group (Table 1).

**Table 1:** Study characteristics of included studies

Author (Year)	Country	Study design	Mean Age (Years)	Sample Size	Material	Follow-up period (Months)	Laparoscopic Surgical Technique	Operating time (Minutes)	Most Common Adverse Event	Key comment
Ho et al. 2015 [14]	Taiwan	Retrospective cohort	Control:53.1 +−13.7 Cases: 53.5 +−15.9	Total: 82 Control:70 Cases: 12	*Biological material*: Surgisis (Porcine) *Synthetic mesh*: Polypropylene	18	TEP	Control: 72.1 +/- 24.3 Cases: 78.9 +/- 23.9	*Recurrence*: Controls: 0 Cases: 5 *Reoperation*: Controls: 0 Cases: 5 *Fever*: Controls: 0 Cases: 1 *Seroma*: Controls: 5 Cases: 3 *Epididymitis*: Controls: 1 Cases:0 *Ileus*: Controls: 0 Cases:1 *Chronic pain*: Controls: 5 Cases:3	SIS mesh in TEP herniorrhaphy is associated with a high recurrence rate, due to slow and inadequate integration of host tissue.
Xue et al. 2024 [19]	China	Multicenter, single-blindedrandomized controlled clinical trial	Control:57.85+/- 14.01 Cases: 58.60 ± 15.55)	Total: 50 Control: 25 Cases: 25	*Biological material*: Novel non-crosslinked from porcine urinary bladder matrix and small intestinal submucosa (UBM/SIS) *Synthetic mesh*: Lightweight, microporous, partially absorbable	48	TAPP	Control: 50.92 +/- 24.94 Cases: 53.28 +/- 20.62	*Recurrence*: Controls: 2 Cases: 0 *Reoperation*: Controls: 1 Cases: 0 *Seroma*: Controls: 2 Cases:0 *Discomfort*: Controls:3 Cases: 0 *Surgical site infection* Controls:1 Cases: 0	Biologics have the same efficacy and safety as the synthetic lightweight meshes, as well as good biocompatibility, can reduce postoperative chronic pain, and induce tissue regeneration to strengthen the abdominal wall and avoid hernia recurrence.
Cuihong et al. 2025 ​ [20]​	China	Retrospective cohort	Control: 55.7 ± 14.4 Cases: 30.0 ± 8.5	Total: 1240 Control: 1097 Cases: 143	*Biological material*: Surgisis (Porcine) *Synthetic mesh*: Polypropylene	84.2	TAPP	Control: 50.1 +/- 17.6 Cases: 63.2 +/- 18.6	*Recurrence*: Controls: 0 Cases: 5 *Reoperation*: Controls: 0 Cases: 5 *Discomfort*: Controls: 1 Cases: 10	A 3% of patients were lost to follow-up. The SIS mesh in TAPP hernia repair leads to more frequent discomfort and a higher recurrence rate (not statistically significant) compared to PP.

All studies were assessed as low risk of bias. *UBM/SIS* Porcine urinary bladder matrix; *TAPP* Transabdominal preperitoneal repair; *TEP* Total extraperitoneal repair; *SIS* Small intestinal submucosal mesh; *PP* Polypropylene

Overall, SIS mesh use was associated with a modest increase in operating time compared to controls across studies ​ [20, 21]​, while only one study reported a slightly longer hospital stay for SIS patients ​ [21]​. Recurrence rates were higher in the SIS group in two out of three studies ​ [20, 21]​, though one study reported no recurrences in the SIS group while observing some in the control group ​ [19]​. Complication rates varied, with seromas, foreign body discomfort, and groin pain being the most frequent, while isolated cases of infection, fever, ileus, and epididymitis were reported. Notably, one study indicated more complications in the SIS group ​ [21]​, one study reported more in the control group ​ [20]​, and one found no complications in the SIS group​ [19–21]​.

### Risk of bias

**Fig. 2:**
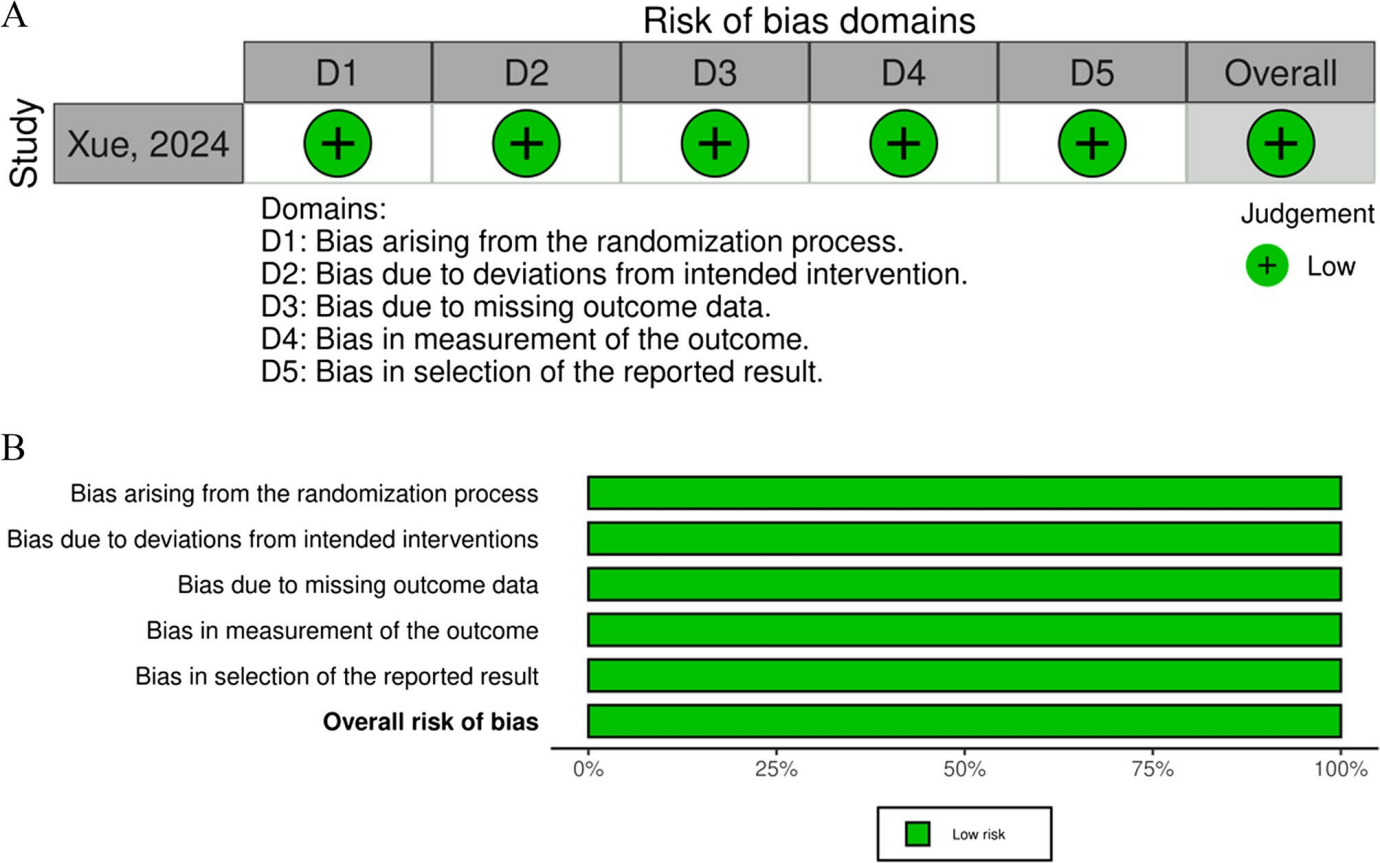
Risk of Bias. Risk of Bias assessment of the included randomized controlled trials using the Cochrane RoB 2.0 tool. **A** Risk of bias by domain for each study and (**B**) overall risk of bias presented as a percentage. Among the seven studies assessed, almost 50% raised some concerns, while 43% had a low risk of bias. No studies were classified as high risk

The observational studies were assessed using the Newcastle-Ottawa Scale​ [10]​, while the RCT was evaluated with the Cochrane RoB 2.0 tool ​ [9]​. The quality assessments yielded the following results: Xue et al. (2024)​ [19]​, low risk of bias (Cochrane RoB 2.0), Cuihong et al. (2025)​ [20]​, high quality (Newcastle-Ottawa Score: 7/9), and Ho et al. (2015) ​ [21]​, high quality (Newcastle-Ottawa Score: 8/9). Overall, all three studies were of high quality and at low risk of bias (Fig. 2A, B and Table 2).

**Table 2:** Risk of bias with NOS

Study	Design	Selection	Comparability	Outcome/Exposure	Total	Overall
Chen-Hsun, 2015	Cohort	☆☆☆☆	☆☆	☆☆☆	9	Low Risk
Cuihong, 2025 [20]	Cohort	☆☆☆☆	☆	☆☆☆	8	Low Risk

Study quality was assessed using the Newcastle-Ottawa Scale (NOS) for cohort studies. Scores are based on three domains: Selection (maximum 4 stars), Comparability (maximum 2 stars), and Outcome/Exposure (maximum 3 stars). A total score of 7–9 indicates low risk of bias, 4–6 indicates moderate risk, and 0–3 indicates high risk

### Meta analysis

#### Recurrence

**Fig. 3:**
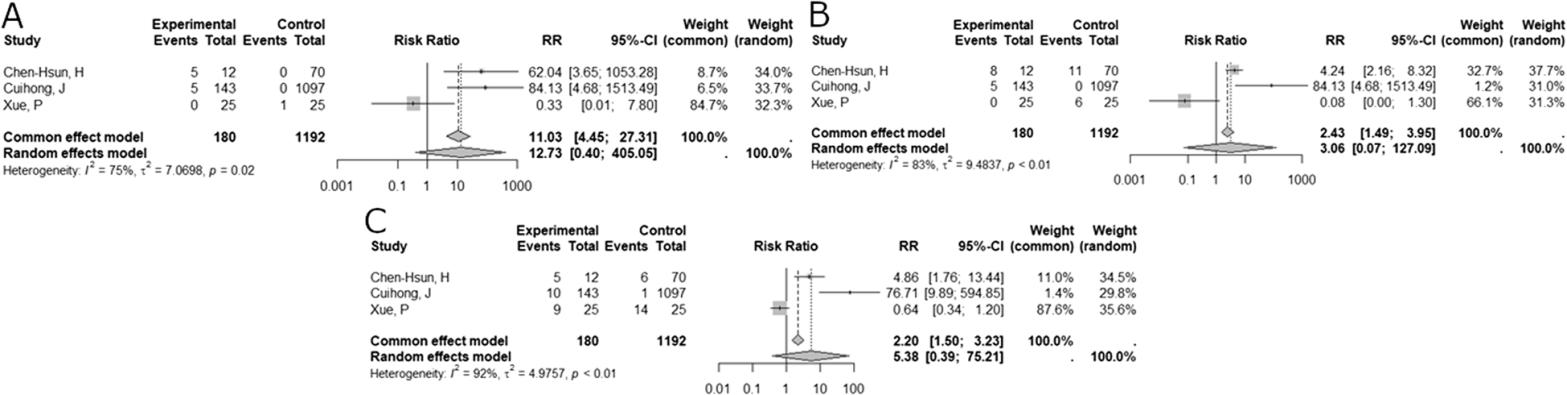
Forest plot of risk ratios. **A**, forest plot of risk ratios (RR) and 95% confidence intervals (CI) for recurrence rates in studies comparing biological versus synthetic mesh. **B,** Forest plot of risk ratios (RR) and 95% confidence intervals (CI) for complications in studies comparing biological versus synthetic mesh. **C**, forest plot of risk ratios (RR) and 95% confidence intervals (CI) for adverse event rates in studies comparing biological versus synthetic mesh

**Fig. 4:**
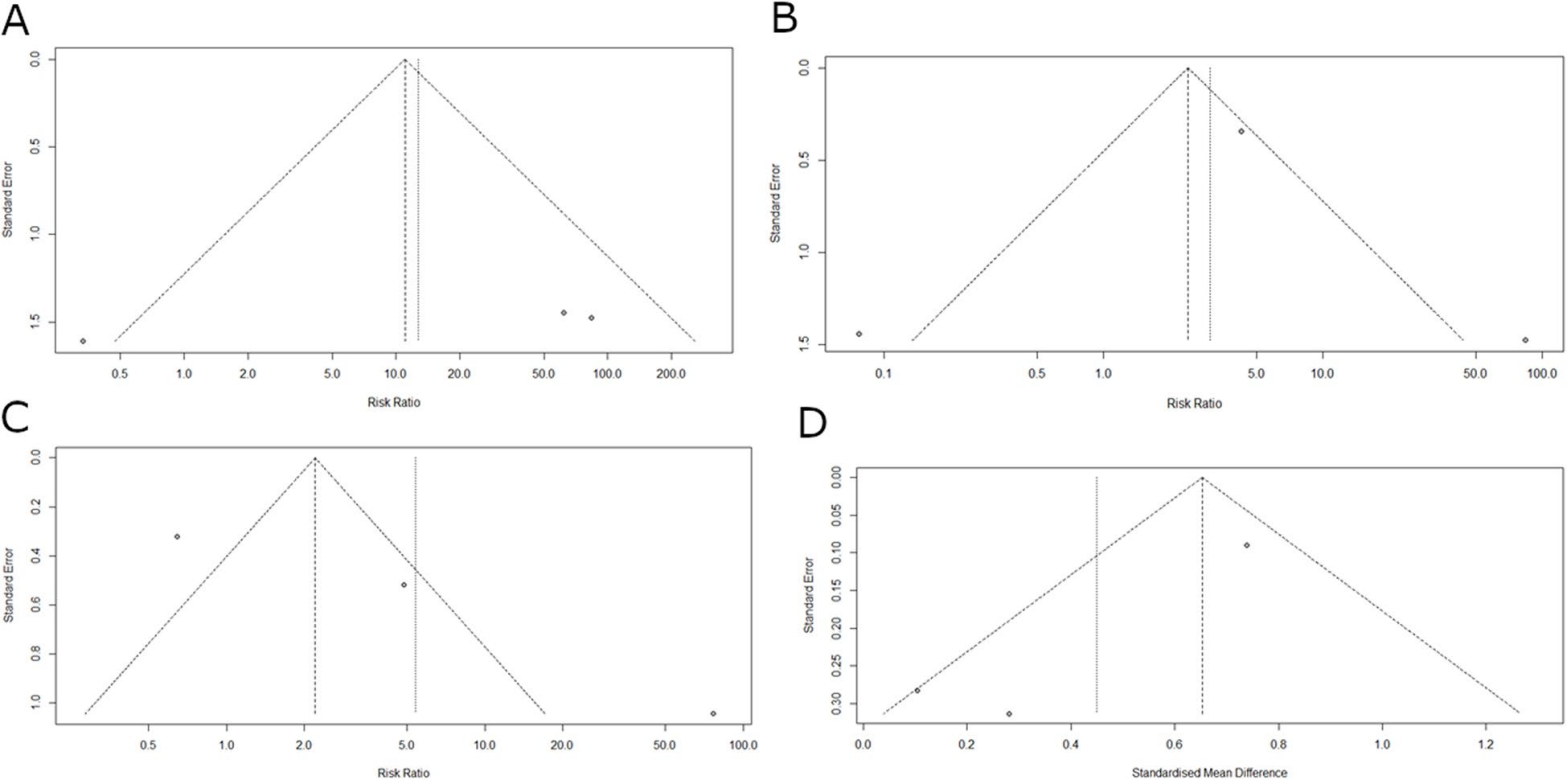
Funnel plot illustrating potential publication bias **A**, funnel plot illustrating potential publication bias in recurrence rates among studies comparing biological versus synthetic mesh. **B,** funnel plot illustrating potential publication bias for complications among studies comparing biological versus synthetic mesh. **C**, funnel plot illustrating potential publication bias for adverse events among studies comparing biological versus synthetic mesh. **D**, funnel plot illustrating potential publication bias for operating times among studies comparing biological versus synthetic mesh

We performed a meta-analysis to compare biological versus synthetic mesh for inguinal hernia. Three studies, with a total of 1372 participants, were included. The pooled analysis indicated a Risk Ratio (RR) of 12.73 (95% CI: 0.40; 405.05, *p*=0.15, I2 = 75%; Fig. 3A). A funnel plot showed visual asymmetry between the studies, indicating potential publication bias. Egger's test for small-study effects was not possible due to the small number of studies (*n* = 3). Sensitivity and subgroup analyses were not performed due to the low number of studies included (Fig. 4A).

#### Complication

We performed a meta-analysis to compare the complications associated with biological versus synthetic mesh for inguinal hernia. Three studies, with a total of 1372 participants, were included. The pooled analysis under the random effects model indicated a Risk Ratio (RR) 3.06 (95% CI: 0.07; 127.09, *p*=0.55, I2 = 83%; Fig. 3B). A funnel plot showed visual asymmetry between the studies, indicating potential publication bias. Egger's test for small-study effects was not possible due to the small number of studies (*n* = 3). Sensitivity and subgroup analyses were not performed due to the low number of studies included (Fig. 4B).

#### Adverse event

We performed a meta-analysis to compare the rate of adverse events associated with the use of biological versus synthetic mesh for inguinal hernia. Three studies, with a total of 1372 participants, were included. The pooled analysis under the random effects model indicated a Risk Ratio (RR) 5.38 (95% CI: 0.39; 75.21, *p*=0.21, I2 = 92%; Fig. 3C). A funnel plot showed visual asymmetry between the studies, indicating potential publication bias. Egger's test for small-study effects was not possible due to the small number of studies (*n* = 3). Sensitivity and subgroup analyses were not performed due to the low number of studies included (Fig. 4C).

#### Operating time

**Fig. 5:**
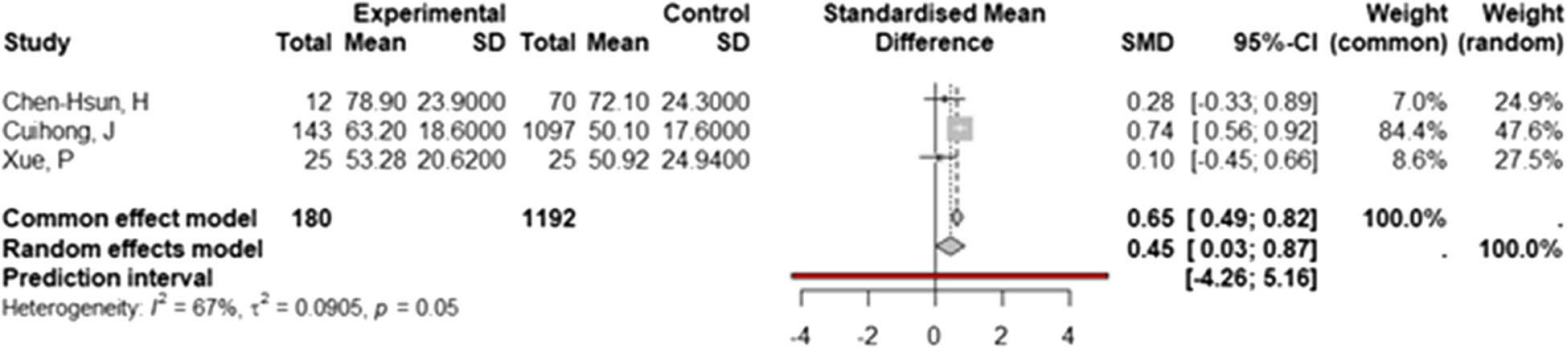
Forest Plot of Operating Time: Biological vs. Synthetic Mesh Forest plot of Standardized Mean Difference (SMD) and 95% confidence intervals (CI) for operating time (in minutes) in studies comparing biological versus synthetic mesh

We performed a meta-analysis to compare the operating time with biological versus synthetic mesh for inguinal hernia. Three studies, with a total of 1372 participants, were included. The pooled analysis using a random-effects model yielded a standardized mean difference (SMD) of 0.45 (95% CI: 0.02–0.87, *p*= 0.039, *I²* = 67%). These results indicate a statistically significant difference in operating time between the two approaches, with procedures involving biological meshes requiring more time than those using synthetic meshes (Fig. 5). A funnel plot showed visual asymmetry between the studies, indicating potential publication bias (Fig. 4D). Egger's test for small-study effects was not possible due to the small number of studies (*n*= 3). Sensitivity and subgroup analyses were not performed due to the low number of studies included.

## Discussion

This systematic review and meta-analysis examined the comparative outcomes of SIS versus synthetic mesh in laparoscopic inguinal hernia repair. Our findings suggest no statistically significant difference between the two mesh types regarding recurrence rates, overall complications, or adverse events. Reported issues such as seroma formation, groin pain, and foreign body discomfort occurred at similar frequencies across both groups. However, a notable difference was observed in operative times, with procedures involving SIS mesh taking longer on average.

Biological mesh has demonstrated benefits in contaminated or infected fields, but its long-term utility in clean cases like laparoscopic inguinal hernia repair remains unclear ​ [22]​. Synthetic mesh, in contrast, has decades of supporting evidence and remains the standard of care in most settings​ [23]​. Previous meta-analyses have largely focused on open surgical approaches, with the most recent in 2015, excluding laparoscopic data ​ [24]​. Given the increasing preference for minimally invasive techniques, this review provides an important update and contributes to evolving surgical decision-making. Overall, our findings align with earlier studies suggesting wide variability in outcomes associated with SIS mesh and reinforce the need for more robust, well-powered randomized controlled trials.

Adverse events such as seromas, foreign body discomfort, and groin pain were noted. Although some studies have proposed that biological mesh may reduce chronic pain, our review did not reveal statistically significant differences in this outcome between mesh types. The inconsistency in pain reporting across studies limited our ability to draw definitive conclusions. However, operative times were statistically significant, with biological mesh leading to prolonged surgical durations, which may be relevant when weighing efficiency against potential benefits. These results closely align with findings from previous studies, such as Fang et al. (2015), who concluded that biological mesh provided no superiority over synthetic mesh ​ [24]​. Our study reinforces these conclusions specifically for laparoscopic repair. Bochicchio et al. (2014) studied predominantly male adults undergoing elective, non-contaminated open inguinal hernia repair and found no significant differences in recurrence or complication rates between biological and synthetic mesh at one-year follow-up [25]​. They emphasized that surgeon experience was a key factor influencing outcomes. Similarly, Bellows et al. (2011) found that biological mesh exhibited comparable intraoperative and early postoperative morbidity to synthetic mesh in a cohort of adult patients, primarily male, undergoing elective inguinal hernia repair in clean surgical conditions ​ [26]​. The study found no significant differences in early complications, including infection, hematoma, or seroma formation, suggesting that biological mesh can be used safely in selected non-contaminated cases without increasing short-term morbidity. Both studies underscore the importance of surgical technique and patient selection, aligning with our findings. However, our review also highlights that SIS mesh may lead to longer operative times.

Although mesh infection was infrequent, its occurrence, even in isolated cases, warrants concern due to its potential severity and need for mesh explanations. Addressing these uncertainties will require future research that reduces heterogeneity through more clearly defined subgroup analyses, which could help clarify potential differences. Expanding databases and increasing sample sizes would mitigate publication bias, while standardizing surgical protocols and mesh application techniques would improve comparability across studies. The length of follow-up plays a critical role in evaluating the true efficacy and safety of mesh implants. In our review, follow-up ranged from 18 to 84.2 months, with two studies providing long-term data. This heterogeneity may influence the reliability of recurrence and complication rates reported. Studies with shorter follow-up durations might underestimate late-onset mesh infections, chronic pain, or recurrences, which can manifest years after surgery. Therefore, future studies should prioritize extended follow-up to capture long-term outcomes accurately, fully assess the durability of biological mesh and determine whether its clinical advantages justify its costs and prolonged operative times.

### Limitations and practical implications

Only three studies met the inclusion criteria ​[19​​, 20,​​ 21]​, limiting the available data and preventing the sensitivity and subgroup analysis. The exclusion of non-English language articles may have introduced language bias and restricted the comprehensiveness of the review. High heterogeneity among studies indicated considerable variability, making it challenging to draw firm conclusions. The complications reporting was inconsistent through the studies, limiting the availability of direct comparisons. Additionally, the funnel plots suggested potential publication bias, but Egger’s test could not be performed to confirm this. Despite these challenges, the fact that there are no previous meta-analyses on this topic highlights novelty and relevance of this study, as it provides a foundational synthesis of available evidence and identifies key gaps to guide future research.

It should be considered that SIS mesh does not demonstrate significant advantages over synthetic mesh in laparoscopic inguinal hernia repair for non-contaminated cases, and synthetic mesh should remain as the primary option with the current information, ensuring both efficiency and durability.

## Conclusion

This systematic review and meta-analysis found no significant differences in recurrence, complications, or adverse events. SIS mesh was associated with longer operative times, also, concerns remain regarding its durability, surgical complexity, and cost-effectiveness. However, no universal recommendation can be made. Mesh selection should consider clinical context and surgeon experience. Further research should focus on larger studies, long-term follow-up, standardized outcomes, and cost-effectiveness analysis.

## Supplementary Information

Supplementary Material 1.

## Data Availability

Data is provided within the manuscript or supplementary information files.

## Abbreviations

NOS: Newcastle-Ottawa Scale
PP: Polypropylene
RCTs: Randomized Control Trials
SIS: Small Intestinal Submucosa
SMD: Standardized Means Difference
TAPP: Transabdominal Preperitoneal Repair
TEP: Total Extraperitoneal Repair
UBM/SIS: Porcine Urinary Bladder Matrix/Small Intestinal Submucosa
