# Correction: Comparative outcomes of synthetic and biological mesh use in laparoscopic inguinal hernia repair: a systematic review and meta-analysis

**DOI:** 10.1186/s12893-025-03308-7

**Published:** 2025-12-19

**Authors:** Candela Romano, Hugo Silva, Laura A. Gray, Carla Ibarra, William Soto, Lorenzo G. Fernandez, Jorge Vazquez del Real, Rafael Pinto-Colmenarez, Victor Sebastian Arruarana, Daniela Fulginiti

**Affiliations:** 1 University of California, Irvine, 101 The City Drive South, ZC4482, Orange, USA; 2 Universidad Autónoma de Baja California, Mexicali, Baja California México; 3 Universidad Autónoma de Guadalajara, Tabasco, México; 4 Pontificia Universidad Católica del Ecuador, Quito, Ecuador; 5 Instituto Tecnológico de Santo Domingo, Santo Domingo, Dominican Republic; 6 Universidad Nacional de Córdoba, Córdoba, Argentina; 7 Universidad de Guadalajara, Guadalajara, Jalisco México; 8 Department of Ophthalmology, University of North Carolina at Chapel Hill, Chapel Hill, USA; 9 Brookdale Hospital, New York, NY USA; 10 Universidad Católica de Argentina, Buenos Aires, Argentina

**Correction: BMC Surg 25, 458 (2025)**

**https://doi.org/10.1186/s12893-025-03151-w**

In this article [1], Figs. 3, 4 and 5 appeared incorrectly and have now been corrected in the original publication. For completeness and transparency, the old incorrect and correct versions are displayed below. The original article has been corrected.

Incorrect Fig. 3:

**Fig. 3:**
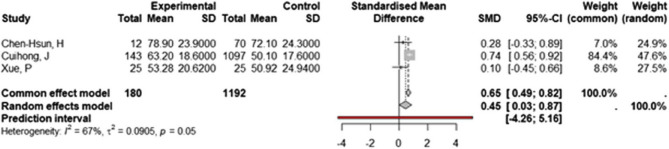
Forest plot of risk ratios. A, forest plot of risk ratios (RR) and 95% confidence intervals (CI) for recurrence rates in studies comparing biological versus synthetic mesh. B, Forest plot of risk ratios (RR) and 95% confidence intervals (CI) for complications in studies comparing biological versus synthetic mesh. C, forest plot of risk ratios (RR) and 95% confidence intervals (CI) for adverse event rates in studies comparing biological versus synthetic mesh

Incorrect Fig. 4:

**Fig. 4:**
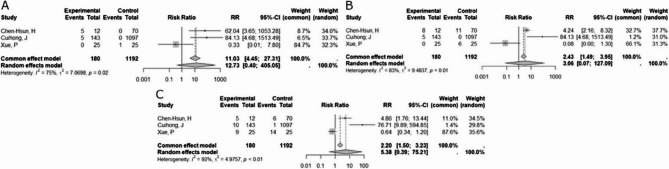
Funnel plot illustrating potential publication bias A, funnel plot illustrating potential publication bias in recurrence rates among studies comparing biological versus synthetic mesh. B, funnel plot illustrating potential publication bias for complications among studies comparing biological versus synthetic mesh. C, funnel plot illustrating potential publication bias for adverse events among studies comparing biological versus synthetic mesh. D, funnel plot illustrating potential publication bias for operating times among studies comparing biological versus synthetic mesh

Incorrect Fig. 5:

**Fig. 5:**
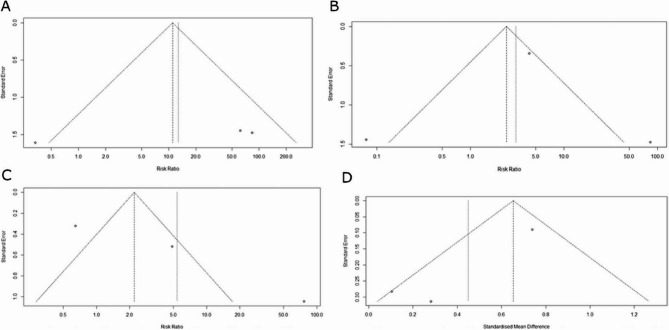
Forest Plot of Operating Time: Biological vs. Synthetic Mesh Forest plot of Standardized Mean Difference (SMD) and 95% confidence intervals (CI) for operating time (in minutes) in studies comparing biological versus synthetic mesh

Correct Fig. 3:

**Fig. 3:**
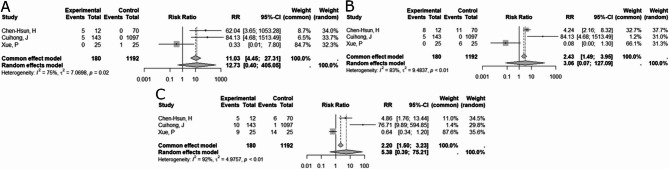
Forest plot of risk ratios. A, forest plot of risk ratios (RR) and 95% confidence intervals (CI) for recurrence rates in studies comparing biological versus synthetic mesh. B, Forest plot of risk ratios (RR) and 95% confidence intervals (CI) for complications in studies comparing biological versus synthetic mesh. C, forest plot of risk ratios (RR) and 95% confidence intervals (CI) for adverse event rates in studies comparing biological versus synthetic mesh

Correct Fig. 4:

**Fig. 4:**
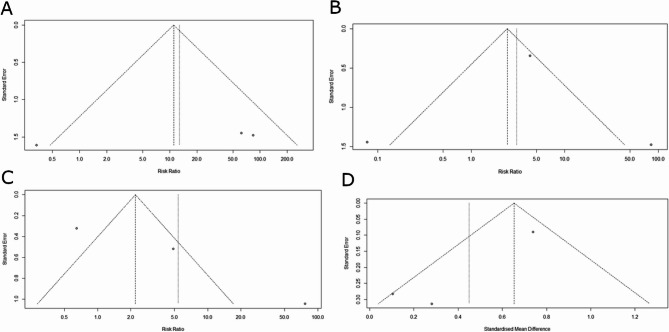
Funnel plot illustrating potential publication bias A, funnel plot illustrating potential publication bias in recurrence rates among studies comparing biological versus synthetic mesh. B, funnel plot illustrating potential publication bias for complications among studies comparing biological versus synthetic mesh. C, funnel plot illustrating potential publication bias for adverse events among studies comparing biological versus synthetic mesh. D, funnel plot illustrating potential publication bias for operating times among studies comparing biological versus synthetic mesh.

Correct Fig. 5:

**Fig. 5:**
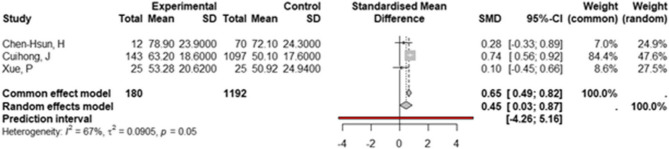
Forest Plot of Operating Time: Biological vs. Synthetic Mesh Forest plot of Standardized Mean Difference (SMD) and 95% confidence intervals (CI) for operating time (in minutes) in studies comparing biological versus synthetic mesh.
