# Supplementary material for: Comparative outcomes of synthetic and biological mesh use in laparoscopic inguinal hernia repair: a systematic review and meta-analysis
Source: BMC Surg. 2025 Oct 8;25:458. doi: 10.1186/s12893-025-03151-w (PMC12505747; doi:10.1186/s12893-025-03151-w)
Supplement: Supplementary file 1 — Supplementary Material 1. [file 12893_2025_3151_MOESM1_ESM.docx]

**Supplementary Material**

**Supplementary Table 1:** PubMed searches

| PubMed | |
| --- | --- |
| Search | Results |
| (((Inguinal hernia[Mesh])) OR (Inguinal*[Title/Abstract])) AND ((laparoscopic surgery[Mesh]) OR (laparoscopic*[Title/Abstract])) AND ((surgical mesh[Mesh]) OR (biological mesh*[Title/Abstract]) OR (synthetic mesh*[Title/Abstract]) OR (polypropylene*[Title/Abstract]))) | 1786 |

**Supplementary Table 2.** Web of Science searches

| Web of science | |
| --- | --- |
| Search | Results |
| TS=("inguinal hernia" OR inguinal*)  AND  TS=("laparoscopic surgery" OR laparoscopic*)  AND  TS=("surgical mesh" OR "biological mesh*" OR "synthetic mesh*" OR polypropylene*) | 379 |

**Supplementary Table 3.** Embase searches

| Embase | |
| --- | --- |
| Search | Results |
| ('inguinal hernia'/exp OR inguinal*:ti,ab)  AND  ('laparoscopic surgery'/exp OR laparoscopic*:ti,ab)  AND  ('surgical mesh'/exp OR 'biological mesh*':ti,ab OR 'synthetic mesh*':ti,ab OR polypropylene*:ti,ab) | 1415 |

**Supplementary Table 4.** CINAHL searches

| CINAHL | |
| --- | --- |
| Search | Results |
| (MH "Hernia, Inguinal" OR inguinal*)  AND  (MH "Laparoscopy" OR laparoscopic*)  AND  (MH "Surgical Mesh" OR "biological mesh*" OR "synthetic mesh*" OR polypropylene*) | 365 |

**Supplementary Table 5.** Scopus searches

| Scopus | |
| --- | --- |
| Search | Results |
| TITLE-ABS-KEY("inguinal hernia" OR inguinal*)  AND  TITLE-ABS-KEY("laparoscopic surgery" OR laparoscopic*)  AND  TITLE-ABS-KEY("surgical mesh" OR "biological mesh*" OR "synthetic mesh*" OR polypropylene*) | 1771 |

**Supplementary Table 6.** Cochrane searches

| Cochrane | | |
| --- | --- | --- |
| Search | | Results |
| 1 | MeSH descriptor: [Hernia, Inguinal] explode all trees | 1793 |
| 2 | inguinal*:ti,ab | 5032 |
| 3 | #1 OR #2 | 5239 |
| 4 | MeSH descriptor: [Laparoscopy] explode all trees | 9208 |
| 5 | laparoscopic*:ti,ab | 26791 |
| 6 | #4 OR #5 | 28038 |
| 7 | MeSH descriptor: [Surgical Mesh] explode all trees | 1244 |
| 8 | biological mesh*:ti,ab | 229 |
| 9 | synthetic mesh*:ti,ab | 377 |
| 10 | polypropylene*:ti,ab | 1056 |
| 11 | #7 OR #8 OR #9 OR #10 | 2373 |
| 12 | #3 AND #6 AND #11 | 301 |

**Supplementary Table 7**. Results for Subgroups (Random Effects Model) for recurrence rates for studies comparing biological versus synthetic mesh.

| Subgroup | k | RR | 95% CI | tau² | tau | Q | I² | p-value (subgroup differences) |
| --- | --- | --- | --- | --- | --- | --- | --- | --- |
| Year | | | | | | | | |
| 2015 | 1 | 62.0400 | [3.6543; 1053.2775] | -- | -- | 0.00 | -- | 0.0190 |
| 2025 | 1 | 84.1289 | [4.6764; 1513.4944] | -- | -- | 0.00 | -- |  |
| 2024 | 1 | 0.3333 | [0.0142; 7.8035] | -- | -- | 0.00 | -- |  |
| Country | | | | | | | | |
| Taiwan | 1 | 62.0400 | [3.6543; 1053.2775] | -- | -- | 0.00 | -- | 0.4373 |
| China | 2 | 5.4977 | [0.0243; 1241.4909] | 12.9147 | 3.5937 | 6.42 | 84.4% |  |
| Type of biological mesh | | | | | | | | |
| Surgisis (Porcine) | 2 | 72.0227 | [9.5292; 544.3537] | 0 | 0 | 0.02 | 0.0% | 0.0049 |
| UBM/SIS | 1 | 0.3333 | [0.0142; 7.8035] | -- | -- | 0.00 | -- |  |
| Risk of Bias | | | | | | | | |
| Low | 3 | 12.7335 | [0.4003; 405.0486] | 7.0698 | 2.6589 | 7.93 | 74.8% | -- |
| Surgical Procedure | | | | | | | | |
| TEP | 1 | 62.0400 | [3.6543; 1053.2775] | -- | -- | 0.00 | -- | 0.0190 |
| TAPP | 1 | 84.1289 | [4.6764; 1513.4944] | -- | -- | 0.00 | -- |  |
| TAPP and TEP | 1 | 0.3333 | [0.0142; 7.8035] | -- | -- | 0.00 | -- |  |

**Supplementary Table 8.** Leave-one-out analysis evaluating recurrence rates for studies comparing biological versus synthetic mesh.

| **Author** | **RR** | **Lower** | **Upper** | **I²** | **R student** | **DFFITS** | **Cook.d** | **Cov.r** | **Tau².del** | **QE.del** | **Hat** | **Weight** | **Inf** | **Is.infl** |
| --- | --- | --- | --- | --- | --- | --- | --- | --- | --- | --- | --- | --- | --- | --- |
| **Omitting Chen-Hsun, H** | 5.497655 | 0.02434509 | 1241.4909 | 0.8443304 | 0.5092286 | 0.3717432 | 0.2263974 | 2.4539362 | 12.91469 | 6.42386233 | 0.3402714 | 34.02714 | no |  |
| **Omitting Cuihong, J** | 4.770615 | 0.02848339 | 799.0190 | 0.8288179 | 0.6366492 | 0.4603266 | 0.3093234 | 2.1907836 | 11.31965 | 5.84173102 | 0.3370944 | 33.70944 | no |  |
| **Omitting Xue, P** | 72.022661 | 9.52921614 | 544.3537 | 0.0000000 | -2.8124847 | -1.8961713 | 0.9635422 | 0.3417694 | 0.00000 | 0.02176744 | 0.3226342 | 32.26342 | * | yes |

**Supplementary Table 9**. Results for Subgroups (Random Effects Model) for complications for studies comparing biological versus synthetic mesh.

| **Subgroup** | **k** | **RR** | **95% CI** | **tau²** | **tau** | **Q** | **I²** | **p-value (subgroup differences)** |
| --- | --- | --- | --- | --- | --- | --- | --- | --- |
| Year | | | | | | | | |
| 2015 | 1 | 4.2424 | [2.1620; 8.3247] | -- | -- | 0.00 | -- | 0.0027 |
| 2025 | 1 | 84.1289 | [4.6764; 1513.4944] | -- | -- | 0.00 | -- |  |
| 2024 | 1 | 0.0769 | [0.0046; 1.2952] | -- | -- | 0.00 | -- |  |
| Country | | | | | | | | |
| Taiwan | 1 | 4.2424 | [2.1620; 8.3247] | -- | -- | 0.00 | -- | 0.8828 |
| China | 2 | 2.5261 | [0.0027; 2401.5611] | 22.3564 | 4.7283 | 11.52 | 91.3% |  |
| Type of biological mesh | | | | | | | | |
| Surgisis (Porcine) | 2 | 13.3921 | [0.7757; 231.2157] | 3.3156 | 1.8209 | 3.89 | 74.3% | 0.0117 |
| UBM/SIS | 1 | 0.0769 | [0.0046; 1.2952] | -- | -- | 0.00 | -- |  |
| Risk of Bias | | | | | | | | |
| Low | 3 | 3.0561 | [0.0735; 127.0909] | 9.4837 | 3.0796 | 11.82 | 83.1% | -- |
| Surgical Procedure | | | | | | | | |
| TEP | 1 | 4.2424 | [2.1620; 8.3247] | -- | -- | 0.00 | -- | 0.0027 |
| TAPP | 1 | 84.1289 | [4.6764; 1513.4944] | -- | -- | 0.00 | -- |  |
| TAPP and TEP | 1 | 0.0769 | [0.0046; 1.2952] | -- | -- | 0.00 | -- |  |

**Supplementary Table 10.** Leave-one-out analysis evaluating complications for studies comparing biological versus synthetic mesh.

| **Author** | **RR** | **Lower** | **Upper** | **I²** | **Rstudent** | **DFFITS** | **Cook.d** | **Cov.r** | **Tau².del** | **QE.del** | **Hat** | **Weight** | **Inf** | **Is.infl** |
| --- | --- | --- | --- | --- | --- | --- | --- | --- | --- | --- | --- | --- | --- | --- |
| **Omitting Chen-Hsun, H** | 2.5260590 | 0.002657011 | 2401.56108 | 0.9132116 | 0.08799691 | 0.06545543 | 0.01002821 | 3.3837269 | 22.356425 | 11.522272 | 0.3767399 | 37.67399 | no |  |
| **Omitting Cuihong, J** | 0.7291456 | 0.014750493 | 36.04309 | 0.8635821 | 1.31298362 | 0.85195115 | 0.56766543 | 1.0948688 | 6.943535 | 7.330419 | 0.3103076 | 31.03076 | * | yes |
| **Omitting Xue, P** | 13.3921444 | 0.775680590 | 231.21570 | 0.7431233 | -1.88358580 | -1.13753629 | 0.60349922 | 0.5839667 | 3.315614 | 3.892918 | 0.3129525 | 31.29525 | * | yes |

**Supplementary Table 11**. Results for Subgroups (Random Effects Model) for adverse events for studies comparing biological versus synthetic mesh.

| **Subgroup** | **k** | **RR** | **95% CI** | **tau²** | **tau** | **Q** | **I²** | **p-value (subgroup differences)** |
| --- | --- | --- | --- | --- | --- | --- | --- | --- |
| **Year** | | | | | | | | |
| 2015 | 1 | 4.8611 | [1.7588; 13.4354] | -- | -- | 0.00 | -- | < 0.0001 |
| 2025 | 1 | 76.7133 | [9.8932; 594.8465] | -- | -- | 0.00 | -- |  |
| 2024 | 1 | 0.6429 | [0.3432; 1.2042] | -- | -- | 0.00 | -- |  |
| **Country** | | | | | | | | |
| Taiwan | 1 | 4.8611 | [1.7588; 13.4354] | -- | -- | 0.00 | -- | 0.9139 |
| China | 2 | 6.3322 | [0.0587; 683.6498] | 10.8360 | 3.2918 | 19.14 | 94.8% |  |
| **Type of biological mesh** | | | | | | | | |
| Surgisis (Porcine) | 2 | 16.6349 | [1.1318; 244.5039] | 3.1249 | 1.7677 | 5.59 | 82.1% | 0.0209 |
| UBM/SIS | 1 | 0.6429 | [0.3432; 1.2042] | -- | -- | 0.00 | -- |  |
| **Risk of Bias** | | | | | | | | |
| Low | 3 | 5.3825 | [0.3852; 75.2063] | 4.9757 | 2.2306 | 26.31 | 92.4% | -- |
| **Surgical Procedure** | | | | | | | | |
| TEP | 1 | 4.8611 | [1.7588; 13.4354] | -- | -- | 0.00 | -- |  |
| TAPP | 1 | 76.7133 | [9.8932; 594.8465] | -- | -- | 0.00 | -- |  |
| TAPP and TEP | 1 | 0.6429 | [0.3432; 1.2042] | -- | -- | 0.00 | -- | < 0.0001 |

**Supplementary Table 12.** Leave-one-out analysis evaluating adverse events for studies comparing biological versus synthetic mesh.

| **Author** | **RR** | **Lower** | **Upper** | **I²** | **Rstudent** | **DFFITS** | **Cook.d** | **Cov.r** | **Tau².del** | **QE.del** | **Hat** | **Weight** | **Inf** | **Is.infl** |
| --- | --- | --- | --- | --- | --- | --- | --- | --- | --- | --- | --- | --- | --- | --- |
| **Omitting Chen-Hsun, H** | 6.332229 | 0.05865157 | 683.64976 | 0.9477564 | -0.0644823 | -0.08300257 | 0.01458748 | 3.151921 | 10.836003 | 19.141109 | 0.3451672 | 34.51672 | no |  |
| **Omitting Cuihong, J** | 1.696497 | 0.23400925 | 12.29909 | 0.9092125 | 1.9119047 | 1.23012306 | 0.73637090 | 0.564290 | 1.860673 | 11.014727 | 0.2983480 | 29.83480 | * | yes |
| **Omitting Xue, P** | 16.634913 | 1.13176234 | 244.50393 | 0.8211616 | -1.4394754 | -1.05194747 | 0.70329832 | 1.038775 | 3.124939 | 5.591639 | 0.3564848 | 35.64848 | * | yes |

**Supplementary Table 13.** Results for Subgroups (Random Effects Model) for operating time for studies comparing biological versus synthetic mesh.

| **Subgroup** | **k** | **SMD** | **95% CI** | **tau²** | **tau** | **Q** | **I²** | **p-value (subgroup differences)** |
| --- | --- | --- | --- | --- | --- | --- | --- | --- |
| **Year** | | | | | | | | |
| 2015 | 1 | 0.2778 | [-0.3361; 0.8917] | -- | -- | 0.00 | -- | **0.0464** |
| 2025 | 1 | 0.7389 | [0.5623; 0.9156] | -- | -- | 0.00 | -- |  |
| 2024 | 1 | 0.1015 | [-0.4532; 0.6563] | -- | -- | 0.00 | -- |  |
| **Country** | | | | | | | | |
| Taiwan | 1 | 0.2778 | [-0.3361; 0.8917] | -- | -- | 0.00 | -- | **0.6537** |
| China | 2 | 0.4767 | [-0.1381; 1.0915] | 0.1590 | 0.3988 | 4.60 | 78.3% |  |
| **Type of biological mesh** | | | | | | | | |
| Surgisis (Porcine) | 2 | 0.6060 | [0.1965; 1.0154] | 0.0532 | 0.2306 | 2.00 | 50.0% | **0.1516** |
| UBM/SIS | 1 | 0.1015 | [-0.4532; 0.6563] | -- | -- | 0.00 | -- |  |
| **Risk of Bias** | | | | | | | | |
| Low | 3 | 0.4484 | [0.0227; 0.8741] | 0.0911 | 0.3019 | 6.14 | 67.4% | **--** |
| **Surgical Procedure** | | | | | | | | |
| TEP | 1 | 0.2778 | [-0.3361; 0.8917] | -- | -- | 0.00 | -- | **0.0464** |
| TAPP | 1 | 0.7389 | [0.5623; 0.9156] | -- | -- | 0.00 | -- |  |
| TAPP and TEP | 1 | 0.1015 | [-0.4532; 0.6563] | -- | -- | 0.00 | -- |  |

**Supplementary Table 14.** Leave-one-out analysis evaluating operating times for studies comparing biological versus synthetic mesh.

| **Author** | **Effect** | **Lower** | **Upper** | **I²** | **Rstudent** | **DFFITS** | **Cook.d** | **Cov.r** | **Tau².del** | **QE.del** | **Hat** | **Weight** | **Inf** | **Is.infl** |
| --- | --- | --- | --- | --- | --- | --- | --- | --- | --- | --- | --- | --- | --- | --- |
| **Omitting Chen-Hsun, H** | 0.4766905 | -0.1380827 | 1.0914637 | 0.7828276 | -0.3335171 | -0.1117087 | 0.01695725 | 2.0858532 | 0.15903083 | 4.6046364 | 0.2492621 | 24.92621 | no |  |
| **Omitting Cuihong, J** | 0.1807673 | -0.2308315 | 0.5923662 | 0.0000000 | 2.4423896 | 4.3068492 | 1.51864883 | 0.9349799 | 0.00000000 | 0.1744189 | 0.4752719 | 47.52719 | * | yes |
| **Omitting Xue, P** | 0.6059564 | 0.1965492 | 1.0153636 | 0.5003339 | -1.1992349 | -0.8221673 | 0.52622645 | 0  .9250491 | 0.05318983 | 2.0013365 | 0.2754660 | 27.54660 | * | yes |
